# Supplementary figures and images for: Smoothies Reduce the “Bioaccessibility” of TiO2 (E 171) in the Model of the In Vitro Gastrointestinal Tract
Source: Nutrients. 2022 Aug 25;14(17):3503. doi: 10.3390/nu14173503 (PMC9460534; doi:10.3390/nu14173503)

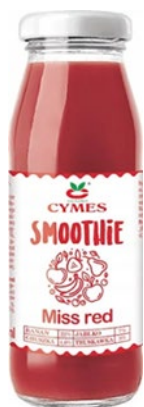

**Figure S1.** Smoothie.

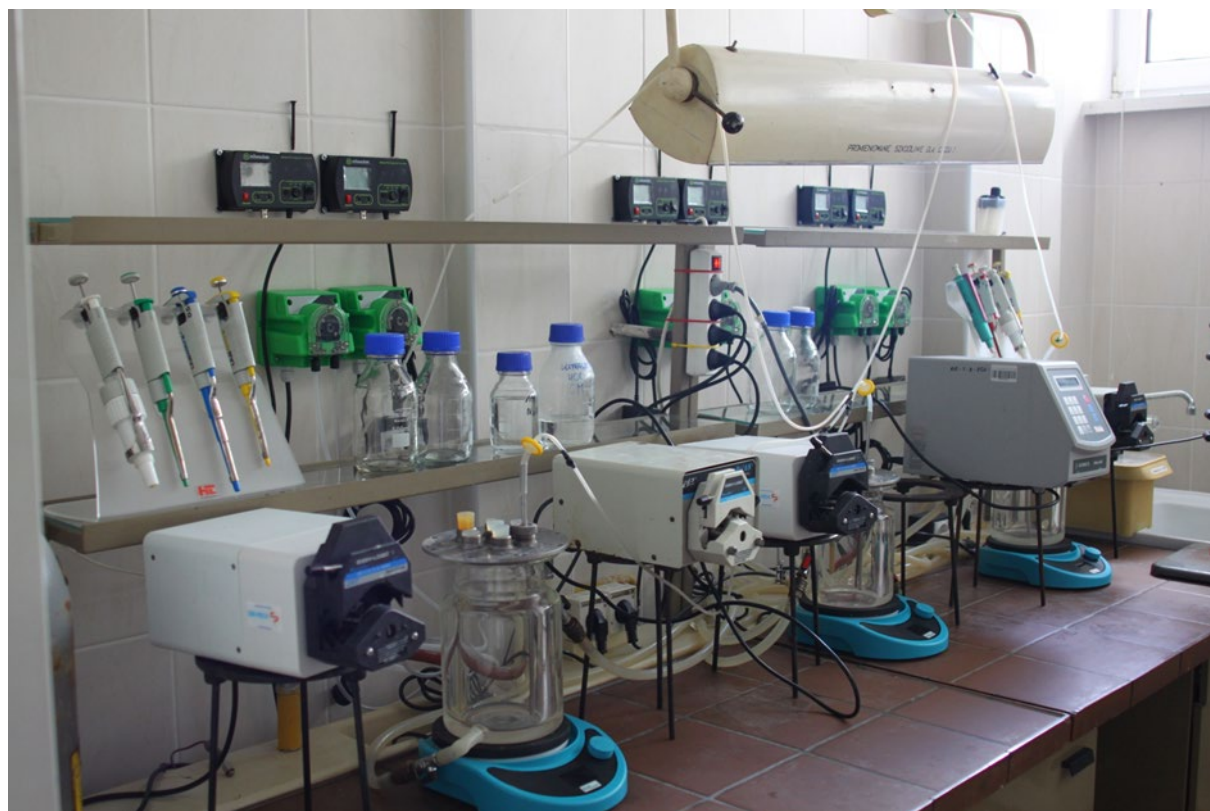

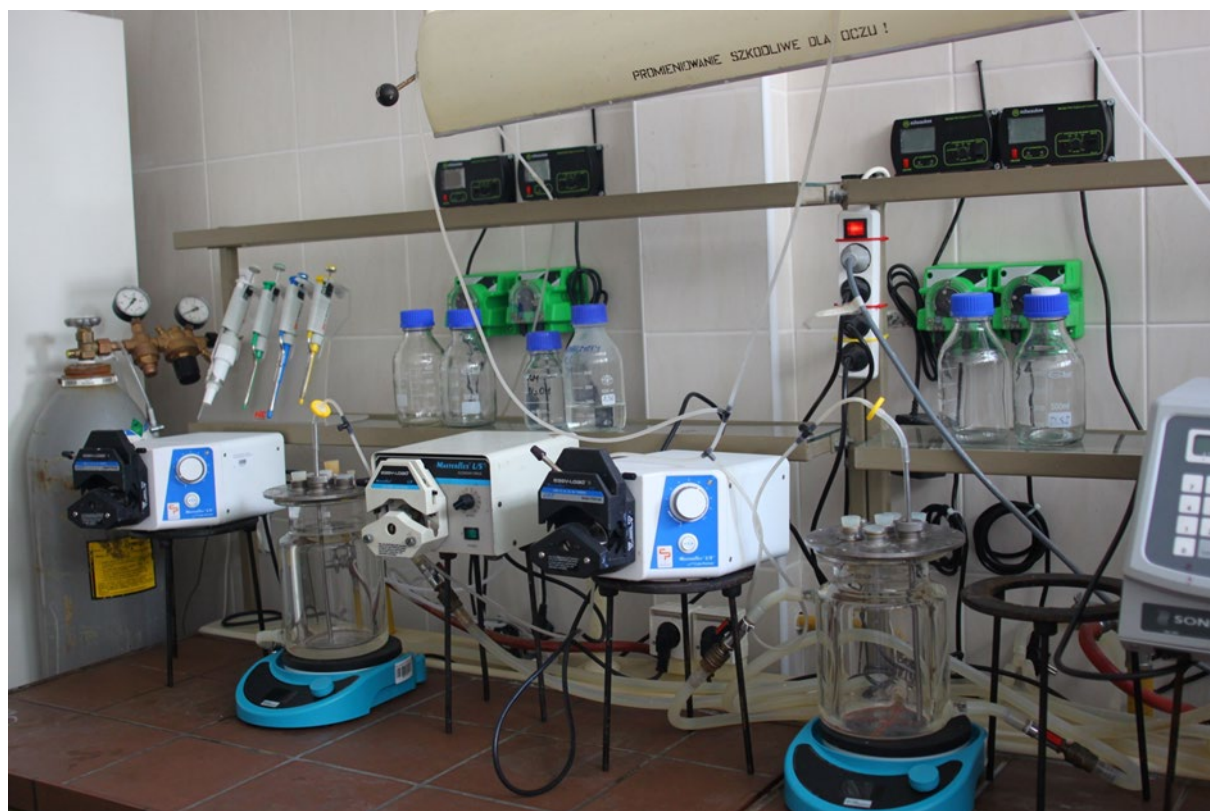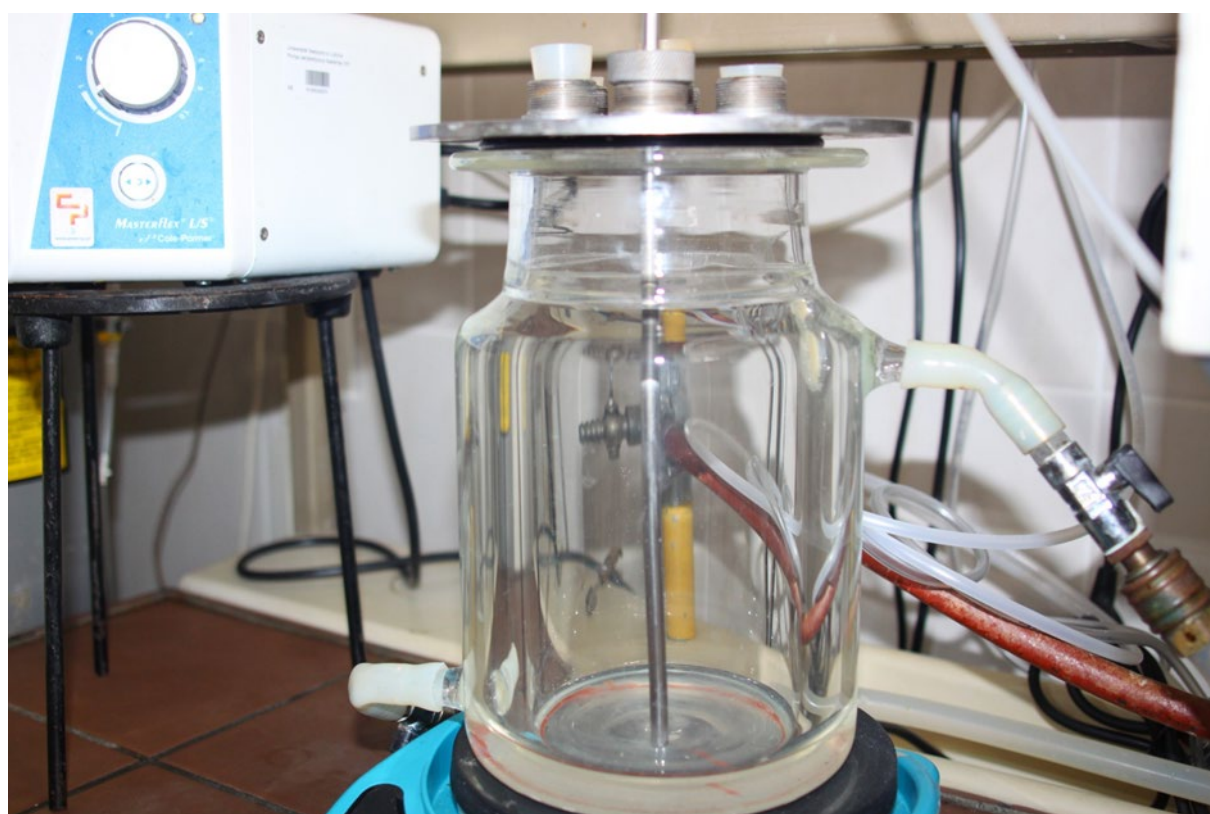

**Figure S2.** *In vitro* model of the “gastrointestinal tract”.

Supplement: Supplementary file 1 [file nutrients-14-03503-s001.zip › nutrients-1863477-supplementary.pdf]
